# The Association Between Cognitive Reserve and Cognitive Trajectories Among Older Adults

**DOI:** 10.1093/geroni/igae014

**Published:** 2024-02-14

**Authors:** Rabia Khalaila, Christina Dintica, Kristine Yaffe

**Affiliations:** Department of Neurology, Global Brain Health Institute, University of California San Francisco, San Francisco, California, USA; Nursing Department, Zefat Academic College, Zefat, Israel; Department of Psychiatry and Behavioral Sciences, University of California at San Francisco, San Francisco, California, USA; Department of Neurology, Global Brain Health Institute, University of California San Francisco, San Francisco, California, USA; Department of Psychiatry and Behavioral Sciences, University of California at San Francisco, San Francisco, California, USA

**Keywords:** Cognitive decline, Composite CR, Individual CR indicator, Longitudinal

## Abstract

**Background and Objectives:**

Prior studies on cognitive reserve (CR) and cognitive trajectories are limited and have had conflicting results. Furthermore, most studies have used a single measure of CR that may not reflect a comprehensive exposure. The objective of this study is to determine the impact of individual and composite CR measures on cognitive decline over a 6-year period.

**Research Design and Methods:**

We studied 55,340 participants from 16 European countries, aged 50 and older, who participated in the Survey of Health, Aging, and Retirement in Europe. We used cognitive measures (including immediate memory, delayed memory, verbal fluency, and numeracy) and 3 CR factors (education, occupation, and cognitive activities) collected in 4 waves from 2011 to 2017. Structural equation modeling was used to construct the composite CR score, analyzed as tertile. Linear mixed-effect models were used to examine the study aims.

**Results:**

At baseline, the highest composite CR tertile was associated with a higher cognition score than the middle and lowest CR tertiles (β: −0.28, 95% confidence interval [CI]: −0.29 to −0.26; β: −0.71, 95% CI: −0.72 to −0.70, respectively), as well as for all individual cognitive domains. At longitudinal results, compared with the lowest CR, the highest but not the middle CR tertile demonstrated a slower 6-year decline in global cognition (β: −0.02, 95 % CI: −0.03 to −0.01), as well as in all cognitive domains (*p* < .05).

**Discussion and Implications:**

A composite CR could be a protective factor for cognitive performance and cognitive decline, and it is more sensitive and inclusive than an individual CR indicator alone.

**Translational Significance:** Cognitive impairment is a major public health concern due to increased life expectancy and aging populations worldwide. High cognitive reserves appear to prevent cognitive decline. Policy-makers and practitioners must suggest strategies to enhance cognitive abilities among older adults, including engaging in cognitively stimulating activities, creating age-friendly workplace policies, offering opportunities for lifelong learning, implementing supportive technology, and designing urban planning strategies that promote cognitive stimulation and social interaction. Additionally, it is important to investigate the impact of cognitive reserve on patients with Alzheimer’s disease and dementia.

Cognitive decline tends to occur with aging and increases the risk of developing mild cognitive impairment (MCI) and dementia (Cheng, 2016; Yates et al., 2016). However, it is still unclear why some older people decline at a faster rate than others (Opdebeeck et al., 2016) and why in some individuals, despite the presence of brain pathology, there may be no cognitive symptoms (Stern, 2002, 2012). A growing body of evidence suggests that cognitive decline and dementia might be delayed with increasing cognitive reserve (CR) throughout the life course (Amanollahi et al., 2021; Dekhtyar et al., 2016; Fritsch et al., 2007; Li et al., 2021).

CR refers to the ability to preserve cognitive function despite the presence of brain pathology or age-related brain changes (Cheng, 2016; Leon et al., 2014; Stern, 2002). The CR theory suggests that attaining an active and cognitively stimulating lifestyle through higher education, higher occupational complexity, and leisure activities can increase CR (Cheng, 2016; Dekhtyar et al., 2016; Fritsch et al., 2007; Leon et al., 2014; Opdebeeck et al., 2016; Stern, 2012; Wilson et al., 2002). CR is hypothesized to increase the brain’s capability for tolerating and compensating for brain atrophy or functional neuronal loss, and consequently, slow down the appearance of clinical manifestations, rate of cognitive decline, and the risk of developing MCI or dementia (Amanollahi et al., 2021; Cheng, 2016; Liu et al., 2013; Stern, 2012; Xu et al., 2020).

Numerous studies of CR have used different measures to assess CR (Li et al., 2021; Opdebeeck et al., 2016; Relander et al., 2021; Xu et al., 2020), most often examining individual measures such as education, occupation complexity, and socially and cognitively stimulating activities (Amanollahi et al., 2021; Liu et al., 2013; Opdebeeck et al., 2016; Weaver & Jaeggi, 2021; Yates et al., 2016), whereas few studies have used a composite CR indicator combining several of these factors. Findings from the cross-sectional results showed consistent positive relationships between composite CR and individual CR indicators with global cognition and cognitive domains (Finkel et al., 2009; Fujishiro et al., 2019; Huang et al., 2020; Li et al., 2021; Mousavi-Nasab et al., 2014; Opdebeeck et al., 2015, 2016; Rydström et al., 2022). However, longitudinal studies are scarce and have had inconsistent results (Lavrencic et al., 2018; Li et al., 2021; Opdebeeck et al., 2016). In one longitudinal study, higher composite CR was associated with better baseline cognitive performance across all cognitive domains, but the association with cognitive decline and incident dementia was not significant (Lavrencic et al., 2018). The use of different individuals and combinations of CR indicators might explain the variability in the results from previous studies (Li et al., 2021; Opdebeeck et al., 2016; Relander et al., 2021; Xu et al., 2020).

The aim of the current study was to examine the associations of both composite CR and individual CR indicators on cross-sectional and longitudinal cognitive performance and cognitive decline over 6 years of follow-up in a large cohort of European older adults.

## Method

### Design and Sample

We used data from the Survey of Health, Aging and Retirement in Europe (SHARE; Börsch-Supan et al., 2013). The survey assessed a representative sample of adults aged 50 and over living in the community, in 10 countries in Europe. The questionnaires were administered using computer-assisted personal interviews. We used data collected in four waves, at 2-year intervals, from 2011 (baseline, Time 1), 2013 (Time 2), 2015 (Time 3), and 2017 (Time 4; Börsch-Supan, 2022). In total, the cohort included 55,340 participants from the following 16 European countries: Germany, Switzerland, Belgium, Poland, Hungary, Spain, Italy, France, Portugal, Czech Republic, Slovenia, Estonia, Austria, Sweden, The Netherlands, and Denmark. Participants were excluded if they had a diagnosis of Alzheimer’s disease or dementia at the baseline wave of the study. Our final cohort consisted of 29,717 participants who provided complete information for baseline and repeat cognitive performance 6 years later (Time 4). We compared these participants to those who were not included in the final analysis due to missing information, using *t*-test analyses for continuous variables and chi-square tests for categorical variables. Participants with missing information in Time 4 were more likely to be female, to be older, to have more chronic diseases and functional dependence, to be less physically active, and to have lower CR measures (see Supplementary Table 1).

### Measures

#### Independent variable

*Cognitive reserve* was operationalized as a combined indicator that included three measures: education level, occupational complexity, and cognitive activities. These factors were chosen as indicative of reserve based on previous work (Kaup et al., 2015; Opdebeeck et al., 2016). Educational attainment was assessed as a 7-point (score 0–6) scale (based on the classification of International Standard Classification of Education—1997): 0 = none, 1 = primary level, 2 = lower secondary, 3 = upper secondary, 4 = postsecondary no tertiary, 5 = first stage of tertiary, and 6 = second stage of tertiary. Occupational complexity was defined based on the International Standard Classification of Occupations (ISCO-08) occupational skill levels with scores ranging from 0 to 4: 0 = unemployed, 1 = simple physical or manual routine tasks (such as elementary occupations), 2 = tasks that require good literacy and numeracy, interpersonal communication skills, or manual dexterity (i.e., service and sales workers), 3 = complex tasks requiring an extensive body of factual, technical, and procedural knowledge in a specialized field (for instance, technicians and associate professionals), 4 = tasks that require complex problem solving and decision making based on extensive theoretical knowledge in a specialized field (i.e., professionals, physicians). Cognitive activities included self-reporting of four kinds of activities: attending an educational or training course, reading books, magazines, or newspapers, doing word or number games such as crossword puzzles, and playing cards or games, with scores ranging from 0 to 4.

Structural equation modeling (SEM) was used to construct the CR composite score based on the best-fitting SEM with the three CR-enhancing factors (education, occupation, and cognitive activity). This method has previously been used to derive CR measures and allows weights to be assigned to each factor in the model (Xu et al., 2020). The final predicted value of the CR for each participant was generated by summing the products of the standardized factor scores and the corresponding SEM factor weights. The weight for each of the four factors was the coefficient of the corresponding factor derived from the SEM with 0.869 for education, 0.602 for occupation, and 0.327 for intellectual activity. The *R*^2^ for education was 0.24, 0.89 for intellectual activity, and 0.64 for occupation. The omega coefficient of the model was 0.65, and internal consistency is considered acceptable if the coefficient is 0.7 or higher. The CR score ranged from −2.826 to 3.781, with a higher score indicating a greater level of CR. The CR score was divided in tertiles: lowest group (−2.826 to −0.6173), middle group (−0.565 to 0.318), and highest group (0.369–3.781).

#### Dependent variable

*Cognitive performance* was measured with four cognitive tests for immediate memory, delayed memory, numeracy, and verbal fluency. These measurements were assessed at baseline and at three subsequent follow-up points (Khalaila et al., 2022; Listl, 2014; Schwartz et al., 2019). The immediate and delayed verbal recall tests were episodic memory tasks that assess short-term verbal learning, memory, and information retention (Cheke & Clayton, 2013). Respondents are asked to recall immediately as many words as possible from a list of 10 words read by the interviewer. Respondents are asked to repeat these words 5–10 min later in the delayed recall task. Scores range from 0 to 10 for each test. To test the numeracy skills (attention function) of the participants, the respondents were asked to subtract 7 from 100, and then continue to subtract from the answer given four more times. Respondents received one point in the numeracy test for each correct answer with scores ranging from 0 to 5. Verbal fluency, a measure of executive function and language ability (Henry et al., 2004), was measured by asking respondents to name as many animals as possible in 1 min, with a maximum score of 40 points. A combined measure of cognition in each wave was constructed by averaging the standardized scores of these four measures. The combined score of cognitive performance ranged from −2.77 to 2.98 at baseline (Time 1), −2.83 to 2.80 at Time 2, −2.92 to 2.85 at Time 3, and −2.75 to 3.18 at Time 4.

#### Covariates

We considered five baseline covariates: age, gender, number of chronic diseases, instrumental activities of daily life (IADL), and physical activity. Information on number of chronic diseases was collected by asking participants whether they had ever been diagnosed with a chronic illness from a list of 14 conditions (e.g., diabetic, hyperlipidemia). Difficulties in IADL included assessing need for help on seven items such as using the telephone and housekeeping (range 0–7 with higher score indicating more dependence). Physical activity in the survey was measured by two separate measures: respondents were asked to answer if they participated in moderate activity and vigorous activity. These two measures were combined into one indicator with two levels: never participating in vigorous or moderate physical activity versus regularly participating or active.

### Data Analyses

To compare the CR groups by baseline characteristics, we used one-way analysis of variance test for continuous variables and chi-square analysis for categorical variables.

The association between the combined CR indicator (tertile of CR) with cognitive performance and cognitive decline was estimated using linear mixed-effect models, with follow-up time as the timescale. The fixed effects included CR, follow-up time, and their interaction. To allow for individual differences at baseline and over time, we included random effects for the intercept and slope for the time in the model.

We next tested the association between the combined CR indicator and performance on each of the cognitive tests cross-sectionally and over time. To further explore the role of each CR component, we conducted mixed-effect models testing the association between each individual CR component and the composite cognitive score level as well as in interaction with time. In all analyses, we adjusted for sex and age, IADL, number of chronic diseases, and physical activity. A one-tail *p* value of <.05 was considered to be statistically significant for all tests. All analyses were completed with STATA version 15.1 and SPSS version 25.

## Results

Among the participants, 58.2% were women and the age ranged from 50 to 99 years (mean age: 70.6, standard deviation [*SD*] = 8.9). The vast majority (91.1%) reported that they were physically active. On average, each participant reported about two chronic diseases (*SD* = 1.5) and low functional disability (IADL, mean = 0.4, *SD* = 1.01).

Table 1 displays the results of the bivariate associations between the baseline variables and CR categories. Compared to the highest CR group, participants with the lowest CR were more likely to be older, female, have a greater number of chronic diseases, and a higher score of functional disability (IADL), and were less likely to be physically active.

**Table 1.** Characteristics of the Study by Composite Cognitive Reserve Categories at Baseline (*N* = 55,340)

Characteristics	Composite cognitive reserve			Test, *p* value
	Lowest (*n* = 17,745)	Middle (*n* = 16,646)	Highest (*n* = 20,949)	
Age	68.59 (10.2)	64.15 (9.4)	63.44 (9.1)	*F* = 1,552.35^***^
Sex				χ^2^ = 430.52^***^
Female	10,105 (35.9)	8,794 (28.5)	11,006 (35.6)	
Male	6,640 (27.2)	7,852 (32.1)	9,943 (40.7)	
Chronic diseases	2.06 (1.6)	1.66 (1.5)	1.54 (1.4)	*F* = 605.64^***^
IADL	0.65 (1.3)	0.29 (0.8)	0.18 (0.6)	*F* = 1,126.97^***^
Physically active				χ^2^ = 1,868.03^***^
Inactive	3471 (55.6)	1,662 (25.2)	1,272 (19.3)	
Active	13,952 (28.8)	14,803 (30.6)	19,609 (40.5)	
Education level	1.33 (0.7)	2.61 (0.6)	4.14 (0.9)	*F* = 57,102.51^***^
Occupation complexity	1.42 (0.5)	1.95 (0.4)	2.94 (0.9)	*F* = 14,857.62^***^
Cognitive activity	1.79 (0.8)	2.32 (0.9)	2.76 (0.9)	*F* = 5,645.37^***^

*Notes*: IADL = instrumental activities of daily life. Cognitive reserve categories: lowest group (−2.826 to −0.617), middle group (−0.565 to 0.318), and highest group (0.369–3.781). Scores range of education level (0–6), occupation complexity (0–4), and cognitive activity (0–4).

^***^ *p* Value <.001.

**Figure 1.**
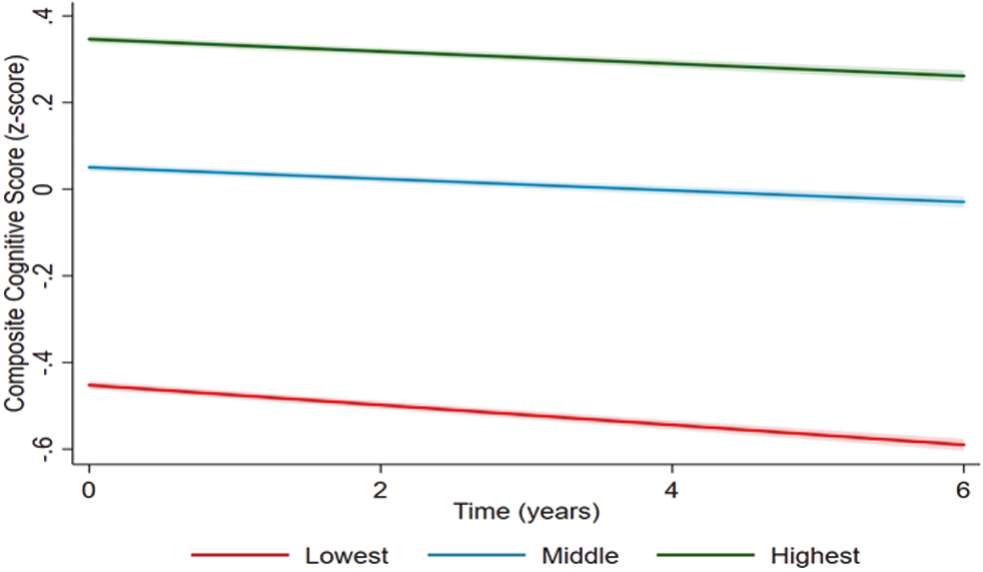
The associations between composite cognitive reserve (tertile) and cognitive decline over 6 years. Adjusted for age and sex, chronic conditions, instrumental activities of daily life, and physical activity.

Table 2 displays the association between the composite CR tertile with combined cognitive performance and cognitive decline in each cognitive domain. At baseline, compared with the highest CR score, participants in the middle and lowest CR tertiles had worse cognitive function in the global cognition score (β: −0.28, 95% CI: −0.29 to −0.26, β: −0.71, 95% CI: −0.72 to −0.70, respectively) and in the four cognitive domains (verbal fluency, numeracy test, immediate memory, and delayed memory). The results also showed that higher composite CR measure had 60%–80% better cognitive performance than lower CR. Moreover, compared to the highest CR, the lowest CR tertile was associated with faster cognitive decline on the combined cognition score as well as on verbal fluency, numeracy test, immediate memory, and delayed memory. The middle CR tertile was not associated with the rate of cognitive decline (Figure 1 and Table 2).

**Table 2.** Associations of Composite Cognitive Reserve (Tertile) With Combined Cognitive Function and Specific Cognitive Domains Over Follow-Up Period

Predictors	Combined cognitive score	β coefficients (95% CI)			
		Verbal fluency	Numeracy	Delayed memory	Immediate memory
*Cognitive reserve*					
Tertile					
Highest	Reference	Reference	Reference	Reference	Reference
Middle	−0.28 (−0.29 to −0.26)	−2.90 (−3.26 to −2.54)	−0.29 (−0.27 to −0.12)	−0.78 (−0.86 to −0.68)	−0.55 (−0.63 to −0.47)
Lowest	−0.71 (−0.72 to −0.70)	−5.53 (−5.88 to −5.18)	−0.89 (−0.97 to −0.82)	−1.38 (−1.48 to −1.29)	−1.05 (−1.13 to −0.98)
*Cognitive reserve × time*					
Tertile					
Highest × time	Reference	Reference	Reference	Reference	Reference
Middle × time	0.00 (−0.00 to 0.01)	0.01 (−0.06 to 0.08)	−0.01 (−0.03 to 0.00)	0.01 (−0.01 to 0.02)	−0.00 (−0.02 to 0.01)
Lowest × time	−0.02 (−0.03 to −0.01)	−0.17 (−0.24 to −0.10)	−0.04 (−0.06 to −0.03)	−0.04 (−0.05 to −0.02)	−0.06 (−0.07 to −0.04)

*Notes*: Models adjusted for age sex, chronic conditions, IADL, and physical activity. CI = confidence interval; IADL = instrumental activities of daily life.

Supplementary Table 2 displays the association of the individual CR indicators (occupation complexity, education, and cognitive activities) with cognitive function. At baseline, a higher score in occupation complexity, education level, and engagement in cognitive activities was individually associated with better composite cognitive performance, and better performance in all cognitive domains. Furthermore, at baseline, the effect of the composite CR score on global cognition and all cognitive domains was stronger than for education alone (β: 0.28, 95% CI: 0.27–0.28; β: 0.17, 95% CI: 0.16–0.18, respectively), but no differences were found with occupation and cognitive activities.

## Discussion

We found that among older adults without dementia, those with lower composite CR had lower scores in the combined cognition score as well as on specific cognitive domains including verbal fluency, numeracy, and memory. Importantly, we showed that the lowest composite CR was associated with faster cognitive decline in both combined and individual cognitive domains. Overall, our results confirm the CR theory hypotheses (Stern, 2012), which suggest that older adults with high CR preserve or improve their cognitive performance and slow cognitive decline, and may also decrease the risk of dementia in later life and help the individual to cope by enlisting compensatory processes (Amanollahi et al., 2021; Cheng, 2016; Li et al., 2021; Liu et al., 2013; Opdebeeck et al., 2015, 2016; Xu et al., 2020). According to these findings, improving CR indicators might lead to better global cognitive performance and may slow the onset of cognitive decline later in life.

A second goal of our study was to examine the association between individual CR indicators and cognitive performance, as well as cognitive decline over a 6-year follow-up. Our baseline cross-sectional results are aligned with the results from previous studies that showed that higher education attainment, greater occupational complexity, and frequent engagement in cognitively stimulating activities were associated with better cognition in the composite cognitive score and in all domains (Fritsch et al., 2007; Huang et al., 2020; Smart et al., 2014; Yaffe et al., 2009; Yates et al., 2016). However, our longitudinal findings showed conflicting results on the association between individual CR indicators and cognitive decline, as found in previous studies (Andel et al., 2006; Li et al., 2021; Lövdén et al., 2020; Mousavi-Nasab et al., 2014; Opdebeeck et al., 2016; Zahodne et al., 2011), although, in line with previous studies, our results showed higher education level is associated with increased cognitive function and low risk of cognitive decline (Dekhtyar et al., 2016; Liu et al., 2013; Opdebeeck et al., 2016; Wang et al., 2023; Yaffe et al., 2009).

Our findings indicated that using composite CR is more inclusive and sensitive at capturing cognitive performance in a combined cognition score and in cognitive domains than using individual CR indicators alone. However, these differences were not found to be significant in the longitudinal findings. These results support those from previous studies which showed that composite CR is more reflective of the individual’s experiences throughout his/her entire lifespan, whereas the individual CR indicator provides a one-point time picture across the lifespan: education mainly reflected early life experience, occupational benefits reflected midlife, and engagement in social and cognitively stimulating activities were expressed in later life (Opdebeeck et al., 2016; Sánchez et al., 2011). For example, one meta-analysis showed that composite CR and education had moderate association with cognitive decline, whereas other individual CR indicators such as cognitively stimulating leisure activities and occupational status had minor associations with cognitive decline (Opdebeeck et al., 2016), or other longitudinal studies that indicated no association between occupational complexity and rates of cognitive decline over time (Lane et al., 2017).

This variability of CR results in our study and in other previous studies could be due to the variations in the measures used to measure individual CR indicators, which makes it difficult to compare between studies. As an example, different studies used different classifications for education attainment versus years of education. As another example, some studies used a combination of occupational complexity measures (such as complexity of work, data, people, and things) instead of individual occupational classifications. Furthermore, different studies used different combinations of leisure and cognitive activities, such as reading books, writing, computer activities, and crossword puzzles. Finally, some studies used two indicators, while others used three or more (Andel et al., 2006; Carlson et al., 2012). Future efforts should aim at generating standardization in CR measurements.

Several limitations of this study should be noted. First, participants of SHARE are generally healthier and more highly educated than the general population, which may overestimate the CR indicators as well as the magnitude of cognitive decline in our study. Second, the cognitive functions used in the study (verbal fluency, numeracy, immediate recall, and delayed recall) are based on simple tests and do not comprehensively reflect overall cognitive function like other known neuropsychological tests. Additionally, the generalizability of our findings is limited to some extent by positive selection due to survival bias of the longitudinal participants in the SHARE cohort. During the follow-up, the survival participants were more likely to be younger, female, healthier, physically active, and with higher scores in CR indicators and cognitive functions. A further limitation may be related to the possibility of reverse causation. It is possible that ongoing cognitive decline may contribute to a reduction in intellectual stimulation. Further, our sample is composed of older adults aged 50 and over; thus, 6-year follow-up might be a limitation. Especially in midlife, longitudinal changes may be too small to detect over time. Moreover, some people may have experienced retirement transitions, such as changing leisure activities or intellectual stimulation. This may have influenced the results. Future studies could use a latent growth curve model based on age to detect shifts around retirement age.

At the same time, the current study has notable strengths. Our study used a community-based cohort with a large sample and long-term follow-up to examine the association of composite CR and cognitive decline. In addition, our results highlight the importance of using a composite CR as a comprehensive CR measure, given that CR is hypothesized to accumulate through lifespan experiences and exposures (Opdebeeck et al., 2016; Stern, 2002; Yates et al., 2016). Our findings tentatively suggest that high engagement in education, occupation, and social activity could strengthen the CR throughout one’s life span, especially in later life with the ultimate goal of preventing cognitive decline, maintaining brain health, and delaying the onset of cognitive impairment and dementia.

## Supplementary Material

igae014_suppl_Supplementary_Table_S1-S2

## Data Availability

This paper uses data from SHARE Waves 4, 5, 6, and 7 (dois: 10.6103/SHARE.w4.800, 10.6103/SHARE.w5.800, 10.6103/SHARE.w6.800, and 10.6103/SHARE.w7.800); see Börsch-Supan et al. (2013) for methodological details.
