# Supplementary material for: The Association Between Cognitive Reserve and Cognitive Trajectories Among Older Adults
Source: Innov Aging. 2024 Feb 14;8(2):igae014. doi: 10.1093/geroni/igae014 (PMC10962634; doi:10.1093/geroni/igae014)
Supplement: igae014_suppl_Supplementary_Table_S1-S2 [file igae014_suppl_supplementary_table_s1-s2.docx]

**Supplementary Table 1**. Descriptive statistics and bivariate analysis comparing participants who were included vs those who were excluded from the final analysis.

| **Variables** | | Included  n= 29,717 | Excluded  n=25,623 | Test | *p*. value |
| --- | --- | --- | --- | --- | --- |
| Gender (%) | Female | 43.9% | 56.1% | χ^2^=164.32 | <0.001 |
|  | Male | 49.3% | 50.7% |  |  |
| Age | Mean (SD) | 64.2 (8.8) | 66.3 (10.7) | t=27.84 | <0.001 |
| Number of chronic diseases | Mean (SD) | 1.7 (1.4) | 1.8 (1.5) | t=9.72 | <0.001 |
| IADL | Mean (SD) | 0.2 (0.7) | 0.5 (1.2) | t=23.03 | <0.001 |
| Physical activity | Active | 56.0% | 44.0% | χ^2^=739.69 | <0.001 |
|  | Inactive | 38.2% | 61.8% |  |  |
| Education level | Mean (SD) | 2.9 (1.4) | 2.7 (1.4) | t=-16.60 | <0.001 |
| Occupation complexity | Mean (SD) | 2.0 (0.9) | 1.9 (0.8) | t=-3.88 | <0.001 |
| Cognitive activity | Mean (SD) | 2.4 (0.9) | 2.3 (0.9) | t=-9.16 | <0.001 |
| Composite CR | Mean (SD) | 0.08 (1.2) | -0.09 (1.2) | t=-16.37 | <0.001 |
| Cognitive performance – Time 4 | Mean (SD) | 0.09 (0.7) | -0.16 (0.8) | t=-37.29 | <0.001 |

*Notes*: IADL (instrumental daily life activities), CR (cognitive reserve).

**Supplementary Table 2**. Associations of the individual CR indicators (continuous indicators) with composite cognitive function and cognitive domains over follow-up period.

| **Predictors** | **β-coefficients (95% CI)** | | | | |
| --- | --- | --- | --- | --- | --- |
|  | Composite cognitive score | Verbal Fluency | Numeracy | Delayed memory | Immediate memory |
| **CR component** |  |  |  |  |  |
| Composite CR | 0.28 (0.27 to 0.28) | 2.00 (1.88 to 2.12) | 0.34 (0.31 to 0.36) | 0.50 (0.47 to 0.53) | 0.40 (0.38 to 0.43) |
| Occupation | 0.27 (0.25 to 0.28) | 2.05 (1.82 to 2.27) | 0.34 (0.29 to 0.39) | 0.49 (0.43 to 0.55) | 0.41 (0.36 to 0.46) |
| Cognitive Activities | 0.24 (0.23 to 0.25) | 2.14 (1.99 to 2.29) | 0.36 (0.33 to 0.39) | 0.45 (0.41 to 0.48) | 0.36 (0.33 to 0.39) |
| Education | 0.17 (0.16 to 0.18) | 1.47 (1.37 to 1.57) | 0.25 (0.23 to 0.27) | 0.39 (0.26 to 0.41) | 0.30 (0.28 to 0.33) |
| **CR component x time** |  |  |  |  |  |
| Composite CR x time | 0.00 (0.00 to 0.01) | 0.05 (0.03 to 0.07) | 0.01 (0.01 to 0.02) | 0.01 (0.00 to 0.01) | 0.01 (0.01 to 0.02) |
| Occupation x time | -0.01 (-0.01 to -0.00) | -0.02 (-0.06 to 0.03) | 0.01 (0.00 to 0.02) | -0.00 (-0.01 to 0.01) | 0.00 (-0.00 to 0.01) |
| Cognitive activities x time | 0.00 (-0.00 to 0.00) | 0.01 (-0.02 to 0.03) | 0.00 (-0.00 to 0.01) | 0.01 (0.00 to 0.02) | 0.01 (0.00 to 0.02) |
| Education x time | 0.01 (0.01 to 0.01) | 0.06 (0.04 to 0.08) | 0.01 (0.01 to 0.02) | 0.01 (0.01 to 0.02) | 0.02 (0.01 to 0.02) |

Notes. Models adjusted for age and sex, chronic conditions, instrumental daily life activities (IADL), and physical activity.
